# Maternal Effects in Relation to Helper Presence in the Cooperatively Breeding Sociable Weaver

**DOI:** 10.1371/journal.pone.0059336

**Published:** 2013-03-25

**Authors:** Matthieu Paquet, Rita Covas, Olivier Chastel, Charline Parenteau, Claire Doutrelant

**Affiliations:** 1 CEFE-CNRS, 1919 Route de Mende, Montpellier, France; 2 CIBIO, Research Centre in Biodiversity and Genetic Resources, Campus Agrário de Vairão, Rua Padre Armando Quintas, Vairão, Portugal; 3 Percy Fitzpatrick Institute, DST/NRF Centre of Excellence, University of Cape Town, Cape Town, South Africa; 4 Biology Department, Science Faculty, University of Porto, Porto, Portugal; 5 Centre d’Études Biologiques de Chizé, CNRS, Villiers-en-Bois, France; Columbia University, United States of America

## Abstract

In egg laying species, breeding females may adjust the allocation of nutrients or other substances into eggs in order to maximise offspring or maternal fitness. Cooperatively breeding species offer a particularly interesting context in which to study maternal allocation because helpers create predictably improved conditions during offspring development. Some recent studies on cooperative species showed that females assisted by helpers produced smaller eggs, as the additional food brought by the helpers appeared to compensate for this reduction in egg size. However, it remains unclear how common this effect might be. Also currently unknown is whether females change egg composition when assisted by helpers. This effect is predicted by current maternal allocation theory, but has not been previously investigated. We studied egg mass and contents in sociable weavers (*Philetairus socius*). We found that egg mass decreased with group size, while fledgling mass did not vary, suggesting that helpers may compensate for the reduced investment in eggs. We found no differences in eggs’ carotenoid contents, but females assisted by helpers produced eggs with lower hormonal content, specifically testosterone, androstenedione (A4) and corticosterone levels. Taken together, these results suggest that the environment created by helpers can influence maternal allocation and potentially offspring phenotypes.

## Introduction

Maximising reproductive success over an individual’s lifetime involves a series of trade-offs between current reproductive effort and survival between breeding events [1]. For females, an important way to adjust the costs of reproduction and influence offspring fitness is the possibility of varying maternal allocation during embryonic development in terms of nutrients, hormones or antibodies [2], [3]. This differential allocation according to early, current or expected environment (e.g. temperature, food availability or mate quality) has been shown in several species [2], [4], .

In egg laying species, differential allocation in reproduction can occur first through the production of eggs of different sizes [7], [8]. Many experiments have demonstrated that egg size is subjected to trade-offs and that these trade-offs change according to the species’ life-history traits and breeding conditions experienced. Larger eggs are more costly to produce [9], [10] but egg size correlates positively with early growth [8], [11]. In particular, several experimental studies on insects, fishes and amphibians, have shown an increase in egg mass in poor environmental conditions, which can be explained by a greater positive effect of egg size on offspring survival under adverse conditions [4], [12], [13], [14]. Similarly, different substances included in the contents of eggs may be submitted to trade-offs between allocation to offspring and mother self-maintenance [15]. For example, carotenoids are fat soluble pigments with antioxidant properties that protect against highly oxidative compounds produced during metabolic and immunological processes [16], [17], [18], [19]. As such, carotenoids are expected to play a central role during embryo development and at hatching [20], [21], [22], but are also important for the breeding female’s own immunity.

Another way through which egg-laying females can alter the environment experienced by their developing offspring is to alter the levels of maternally-derived yolk steroids, such as testosterone and androstenedione (A4), and glucocorticoids, such as corticosterone. In previous studies on birds, androgens (testosterone and A4) were associated with increased begging, growth and early offspring survival ([23] although potentially negative effects on offspring growth and survival have also been reported [24]). Conversely corticosterone is a stress mediated hormone which is assumed to be passively transferred to eggs [25], [26], [27], [28] and overly high corticosterone levels seem mainly detrimental for offspring; reducing hatchling size and growth ([25], [27], [29], [30], [31] but see [32]). Prenatal hormones may also have long-lasting effects on offspring phenotype and fitness such as dispersal behaviour and survival [23], [33], [34], [35]. Hormonal allocation into eggs is strongly influenced by female pre-breeding conditions. For example yolk testosterone deposition in eggs has been found to be influenced by diet quality [36], breeding density and social behavior [37] and females experimentally stressed before laying deposited more corticosterone in their eggs [26], [27], [31].

Maternal allocation in eggs is therefore an epigenetic mechanism influenced by the conditions experienced by the breeding females and by which females can adjust the environment experienced by the developing offspring in order to maximise either offspring or maternal fitness [38]. Previous studies show that the quality of the environment experienced by offspring and environmental predictability are key factors influencing the direction of maternal allocation [12], [13], [39], [40].

Cooperatively breeding species offer a particularly interesting context in which to study maternal allocation and its effects (see also [41]). In these species, sexually mature individuals called ‘helpers’ forgo independent reproduction, but assist the breeders by providing care to their offspring through extra food brought to the nest [42], [43]. Hence, the helpers create predictably improved conditions for offspring development, which is expected to affect female reproductive investment [41]. In addition, cooperative breeders are generally long-lived [44], and hence are expected to favour investment in survival over reproduction. One way in which breeding females may facilitate increased survival is by reducing investment in a current reproductive event when assisted by helpers [45]. For example, it has been shown that parents breeding in groups tend to compensate for the extra food brought by helpers by decreasing their feeding rate (see [46] for a review) which is expected particularly when the costs of egg production are high (Savage et al. in press). This ‘load-lightening’ effect of helpers can also occur through maternal effects.

The first study that investigated this hypothesis was conducted on a cooperatively breeding cichlid and showed that females reduced the size of their eggs according to the experimentally increased number of helpers [47]. In another study on superb fairy wrens *Malurus cyaneus* Russell and co-workers [48], [49] showed that females used the extra food brought by helpers to decrease their own breeding investment. Specifically, these females laid smaller eggs, and experienced improved survival. Nonetheless, the extra food brought by the helpers compensated for the reduction in female investment and hence reproductive output did not differ between nests with and without helpers [48]. However, three additional studies that investigated this possibility obtained contrasting results. There was a similar reduction in egg size in the presence of helpers found in carrion crows *Corvus corone* [50] and southern lapwings *Vanellus chilensis* [51] but no clear support for this hypothesis in acorn woodpeckers *Melanerpes formicivorus* [52]. Hence, studying differential maternal allocation in the presence of helpers is particularly important in order to obtain an understanding of how fitness is maximised in different systems. Simultaneously, it could help explaining puzzling observations from previous studies that detected weak [53], [54] or absent [55], [56] effects of helpers on reproduction. In these species breeding females might save energy in the presence of helpers by producing smaller eggs.

Investigations of egg contents in cooperative breeders are currently needed (see also [41]). Egg size is an important indicator of female energetic investment in reproduction, but more detailed studies of egg contents are required to understand the extent of this investment and the fitness consequences it may have for both mothers and offspring. Russell and collaborators [48] analysed the egg contents in lipids and proteins in superb fairy-wrens *Malurus cyaneus* and found lower levels of these nutrients in the presence of helpers. But to date no study has investigated whether mothers change the allocation of other important egg components such as carotenoids or hormones in relation to helper presence. Hormones, in particular, have a central role in mediating development, competition and sociality and therefore are of particular interest in studies of social and cooperative species [57].

Here, we investigate the effect of helper presence and breeding group size on egg mass and yolk components (carotenoids, testosterone, A4 and corticosterone) of first-laid eggs in a colonial cooperatively breeding bird, the Sociable Weaver, *Philetairus socius*. These weavers are relatively long-lived (the oldest bird recorded was 16 years old and the population average survival rate is 66% [58], although the figure appears to be above 80% for breeders Covas, Deville, Doutrelant and Gregoire unpublished data) and appear to favour investment in survival over reproduction [59]. In agreement with this, parents have been previously shown to reduce their nestling provisioning rates when assisted by helpers and a weak, albeit positive effect of helpers on fledgling mass was found mostly under adverse conditions (i.e. low rainfall or when breeding in large colonies) [54]. Finally, in this species, helpers do not have access to current reproduction and egg dumping has never been observed [60]. Hence, we predicted:

- a reduction in egg size and costly constituents such as carotenoids in presence of helpers;
- an equivalent fledgling mass between nestlings raised with and without helpers, despite initial differences, if helpers compensate for the low maternal investment in eggs the overall feeding rate of a brood was previously found to increase with the number of helpers [54]);
- a differential level of hormones in nests with and without helpers. Based on the positive effects of androgens on early offspring growth found in other studies [23], we expected that eggs laid by females without helpers should have higher levels of androgens to enhance the chicks’ growth, thereby compensating for the lack of help available to raise the offspring (e.g., if nestlings compete more for food in nests without helpers than in nests with helpers and need to be fed more by parents to survive). Finally, corticosterone is thought to be directly related to female stress and likely to be passively transmitted to the eggs [25], [26], [27], [28]. As corticosterone levels are linked with energy expenditure [61], we expected corticosterone levels in eggs to decrease with helper presence if the presence of helpers reduces energy expenditure and stress conditions experienced by females.

## Methods

### Ethics Statements

The work was conducted between September 2010 and February 2011 at Benfontein Nature Reserve in the Northern Cape province of South Africa (28°52′ S, 24°50′E) with the permission of Northern Cape Nature Conservation. The Ethics Committee of the University of Cape Town specifically approved this study (permit number: 5869-2009). De Beers Consolidated Mines provided access to Benfontein Game Reserve.

### Study Species

The sociable weaver is a colonial passerine endemic to the semi-arid acacia savannahs of southern Africa [62], [63]. Sociable weavers build massive communal nests containing several independent nest chambers that are used for breeding and roosting. They are facultative cooperative breeders, breeding in pairs or with up to five helpers (mean group size 3.15 birds for this study, however the proportion of birds breeding in groups varies from ca. 30–80% between years [60]). Helpers are mainly offspring of one or both breeders (93%), although a small number of unrelated birds can also help [60]. Both sexes help, but in a previous study helpers older than one year were found to be only males [64]. [60].

### Field Methods

Before the breeding season 503 individuals roosting in 14 colonies were captured and marked with a unique colour ring combination (see [65] for more details on the captures). Then to determine the onset of reproduction, all nest chambers in these study colonies (i.e. approximately 460) were inspected every 3 days. These chambers were marked with a numbered plastic tag.

As soon as the first eggs were found, colonies were inspected every day in order to mark every new egg laid (with a soft blunt pencil) and thereby know the laying sequence (one egg is laid per day). Sociable weavers usually lay 3–4 eggs (average clutch size is 3.3 [54]). Two days after the first egg in a given nest was laid we weighed all eggs in that clutch to the nearest 0.001 g with a digital Pesola balance (n = 252 eggs from 84 clutches). On this occasion, we collected the first egg laid in that clutch, which was kept frozen until further analyses (n = 84). Only the first egg was collected in order to allow the breeding activity to continue and hence to determine the breeding group size and identity of the individuals feeding at the nest from which we collected an egg. Nest chambers were checked the following day to weigh a possible fourth egg.

To associate every chick with its egg we individually marked 74 hatchlings of 38 clutches (from which we previously collected the first egg) by removing specific down feathers from the neck and/or wings. These marks were still visible 9 days after hatching when the chicks were ringed with a uniquely numbered metal ring. Due to high nest predation by snakes the number of clutches used in this study decreased from the initial 84 to 28 that actually fledged young. We weighed these chicks at 17 days old (46 chicks from 28 clutches).

To identify the individuals feeding at a given chamber and hence the breeding group size, we conducted 1 or 2 hours of daily observations for at least 3 consecutive days (min = 3, max = 10, average = 6.6 days). These observations started when the nestlings were around 6 days old since before the feeding activity is slower. Group size was established when no new birds were seen feeding after on average 5.5 consecutive sessions of observations. Observers were situated in a hide placed at 3–5 m from the colony. We identified 34 breeding groups from chambers where we collected the first egg (18 groups with helpers and 16 without).

Rainfall closely influences food availability and the duration and success of the breeding season in sociable weavers [54], [66], [67]. We therefore monitored rainfall at the study site using a rain gauge.

### Egg Content Assay

Detailed methods of yolk content’s analyses are available in protocol S1. Briefly, fresh yolk carotenoids concentrations were determined by colorimetry [68], [69]. Yolk concentrations of testosterone, A4 and corticosterone were determined by radio-immunoassay [70]. Correlations between first egg mass and the different contents analysed are given in Table 1. As often found in the literature [71], testosterone and A4 concentrations were positively correlated. More surprisingly yolk mass and A4 were negatively correlated (Table 1).

**Table 1:** Pearson correlations and associated p-values between egg mass and components of the first laid eggs.

	yolk mass	carotenoids	Corticosterone	testosterone	androstenedione
egg mass	0.27 (p = 0.13)	0.02 (p = 0.91)	−0.21 (p = 0.23)	0.15 (p = 0.38)	0.01 (p = 0.93)
yolk mass		−0.24 (p = 0.16)	−0.05 p = 0.78)	−0.11 (p = 0.56)	−**0.62 (p = 1 10^−4^)**
carotenoids			0.11 (p = 0.55)	0.15 (p = 0.15)	0.18 (p = 0.32)
corticosterone				*0.33 (p = 0.054)*	0.13 (p = 0.45)
testosterone					**0.46 (p = 0.006)**

### Statistical Analyses

The aim of our analyses was to study the effect of breeding group size or type (with/without helpers) on egg mass, yolk carotenoids and hormonal contents. In addition, we analysed the effect of breeding group on fledging mass taking into account the egg mass. For all these analyses we conducted linear mixed models using the package nlme in R (R Development Core Team, 2011). The final models were obtained by sequentially eliminating explanatory variables with P values >0.1 using a backwards stepwise approach. The minimal model provided the P values of significant terms whereas P values for non-significant terms were obtained by reintroducing each non-significant variable into the minimal model [72].

For each of the following analyses we built two types of models. One using breeding group size as a dependant variable (studying linear and quadratic relationships) and one using breeding group type (i.e. with/without helpers) as the effect of helper presence may be significant but not additive (i.e. regardless of helpers’ number). We present the results based on both group size and group type but as this represents multiple testing we adjusted the P values for false recovery rates [73]. Since, the relatively small sample sizes in this study do not provide strong statistical power, we also discuss the results when they were significant before false recovery rates corrections.

To study the effect of helpers on egg mass we fitted the random factor ‘nest chamber’ in order to take into account the non-independence of eggs from a same clutch. The ‘nest chamber’ factor was nested in a ‘colony’ random factor as we had several nests from each colony. We fitted group size (from 2 to 6 individuals) or group type as a dependant variable and investigated both linear and quadratic relationships for group size. We also added the following co-variables that may affect egg mass in this species [74] and others [7], [8]: laying order (1 to 4); clutch size (2–4); colony size (10–128 individuals); the number of previous breeding attempts in the season (22 eggs were collected during the first breeding attempt and 12 during the second) and rainfall over the 18 days before laying (13.9–94.5 mm). The total rainfall over this period significantly correlated with the number of active clutches (i.e. clutches with eggs or chicks), the number of clutches laid per day and clutch size (Spearman rank correlations, respectively ρ = 0.876; ρ = 0.409; ρ = 0.476 all P<1.2 10^−4^).

For the analyses of yolk mass and contents (i.e. carotenoids and hormones) we included the same terms as above, except ‘nest chamber’ and ‘laying order’ (since we collected only the first egg of each clutch). In addition, we included ‘egg mass of the first egg’ as a fixed term for the analyses of the yolk mass in order to know if the relative investment in yolk differed depending on the presence/number of helpers. As egg and yolk mass of the first eggs collected were not significantly correlated and as both are different indicators of female investment and offspring quality that may be influenced by the mother’s circulating hormones, even independently [75], we included both egg and yolk mass as fixed terms in the analyses of yolk contents. However, as the absolute allocation in yolk mass and contents may be more relevant for offspring fitness, we also present the results without taking into account egg and yolk mass when significant.

In order to investigate the effect of breeding group size and type on fledging mass we used ‘nest chamber’ nested in ‘colony’ as random factors and egg mass, the number of hatchlings, hatching order, colony size, the number of breeding attempts and the rain during the 18 days before laying as fixed factors.

## Results

### Egg Mass

**Figure 1:**
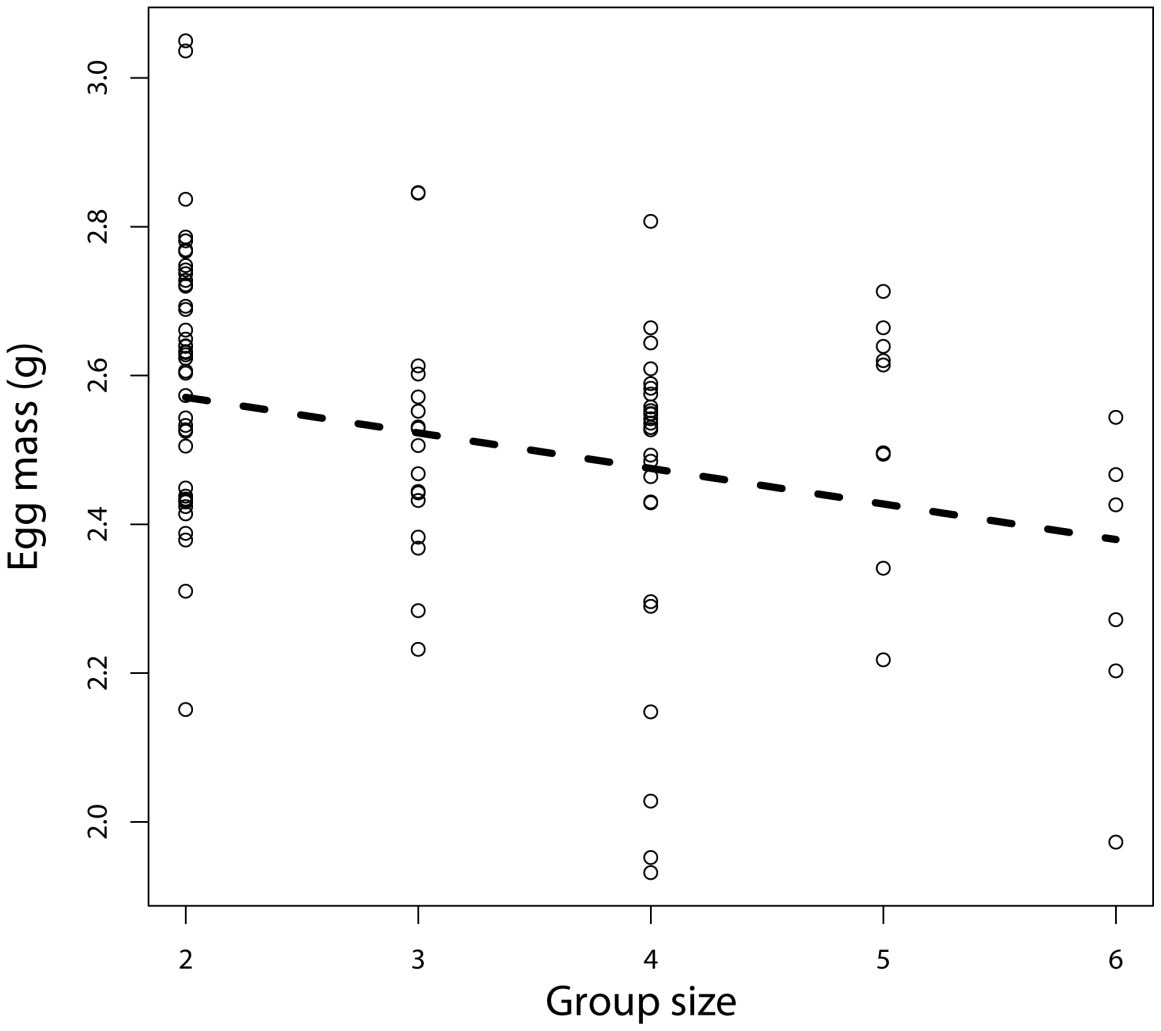
Egg mass decreased in relation to breeding group size. The line indicates the predicted values.

Egg mass varied between 1.932 g and 3.050 g and decreased significantly with the presence of helpers (group type: F_1,23_ = 4.73, P = 0.040, estimate = −0.12±0.055) and helper number (with a linear average decrease of 1.67% per additional helper; Table 2, Fig. 1). In addition, there was a laying order effect, second eggs being significantly heavier than first ones. There was no effect of clutch size, the number of clutches attempted before, colony size or rainfall (Table 2).

**Table 2:** Factors affecting egg mass.

Explanatory terms	F	d.f.	P	Estimate	SE
**Intercept**				**2.666**	**0.082**
**Group size**	**4.82**	**1,23**	**0.04**	−**0.048**	**0.022**
**Laying order**	**4,073**	**3,64**	**0.0104**		
**2**				**0.083**	**0.024**
**3**				**0.036**	**0.025**
**4**				−**0.005**	**0.058**
Group size^2^	0.87	1.22	0.36		
Colony size	2.51	1,8	0.15		
Clutch size	0.36	1,22	0.55		
Breeding attempts	0.22	1,22	0.64		
Rainfall	0.15	1,22	0.70		

Estimates and SE are given for significant explanatory terms included in the minimal model (bold characters). ‘Group size’ referred to the number of individuals that were feeding a given clutch.

### Yolk Mass

**Figure 2:**
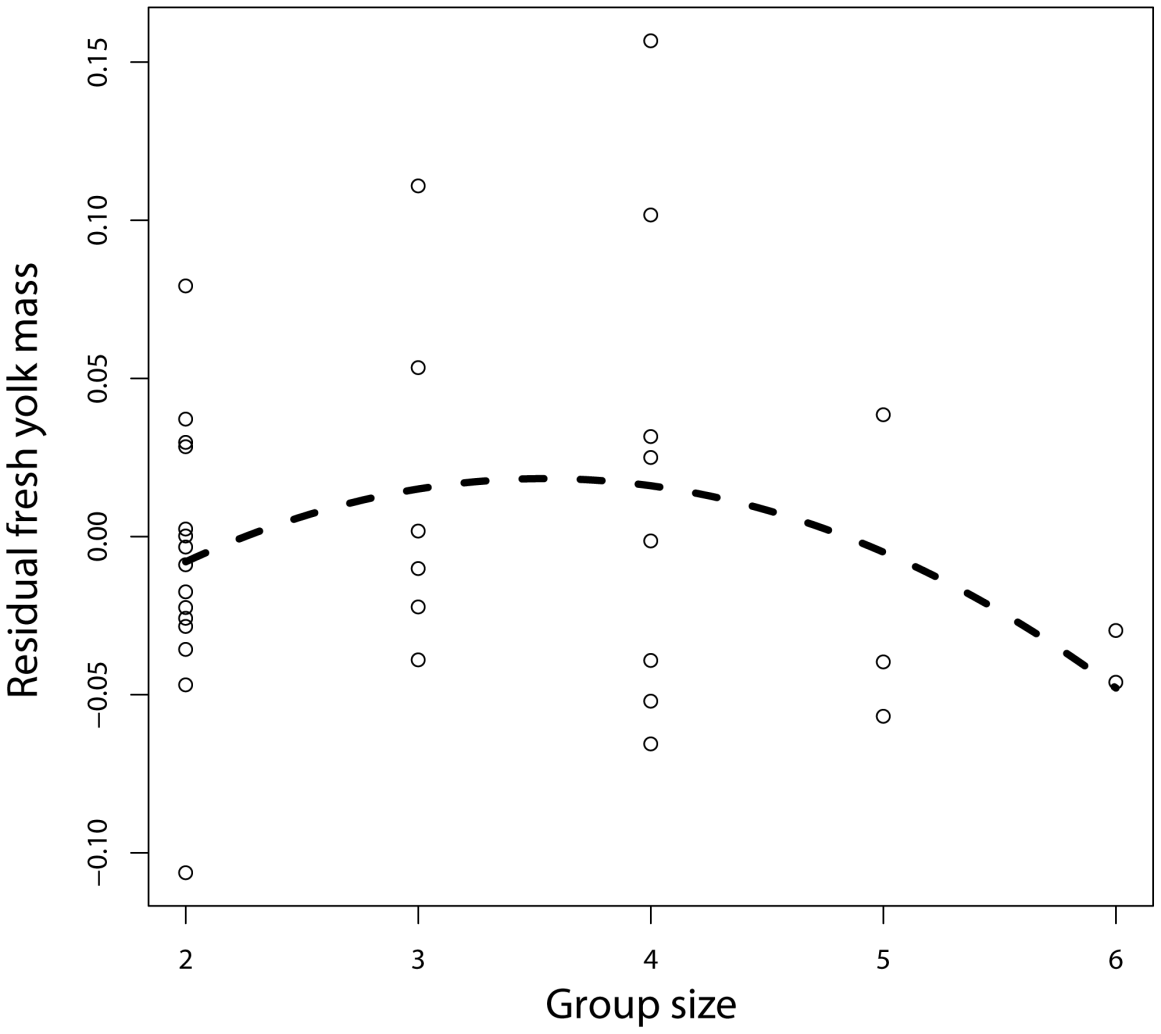
Relationship between fresh yolk mass of first laid eggs (corrected for egg mass) and breeding group size. Dashed line indicates predicted values from the linear mixed-effects model.

The yolk mass of the first egg, ranged between 0.541 g and 0.798 g. It varied in a negative quadratic way with the number of helpers, although this remained below significance (Table 3; Fig. 2; controlling for egg mass). The effect of the number of helpers was not significant without controlling for egg mass (F_1,22_ = 1.84, P = 0.19). Yolk mass was not affected by group type (F_1,22_ = 0.84, P = 0.37), clutch size, the number of clutches attempted before, colony size or rainfall, but increased with egg mass in a linear manner when taking into account the effect of the number of helpers (F_1,21_ = 4.428, P = 0.048, estimate = 0.138±0.066) (Table 3).

**Table 3:** Factors affecting yolk mass.

Explanatory terms	F	d.f.	P	Estimate	SE
Intercept				0.133	0.207
***Group size^2^***	***4.095***	***1,21***	***0.0559***	−***0.014***	***0.007***
***Group size***	***3.938***	***1,21***	***0.0604***	***0.103***	***0.052***
**Egg mass**	**4.428**	**1,21**	**0.0476**	**0.138**	**0.066**
Clutch size	0.002	1,20	0.96		
Clutches attempted before	0.524	1,20	0.48		
Colony size	0.151	1,8	0.71		
Rainfall	0.587	1,20	0.45		

Estimates and SE are given for significant (bold characters) and nearly significant (italic characters) explanatory terms included in the minimal model. ‘Group size’ referred to the number of individuals that were feeding a given clutch.

### Yolk Carotenoids

The concentration of carotenoids varied between 44.69 µg.g^−1^ and 118.80 µg.g^−1^. It was not affected by the number of helpers (linear term, F_1,22_ = 0.081, P = 0.78; quadratic term, F_1,22_ = 0.131, P = 0.72), group type (with or without helpers; F_1,22_ = 0.27 10^−3^, P = 0.96), or any other variable tested (i.e. egg mass, yolk mass, breeding attempts, colony size and rainfall, all P>0.27). There was a tendency for carotenoids to decrease with clutch size, but this was only marginally significant (F_1,23_ = 4.009, P = 0.057, estimate = −13.782±6.883).

### Yolk Androgens

**Figure 3:**
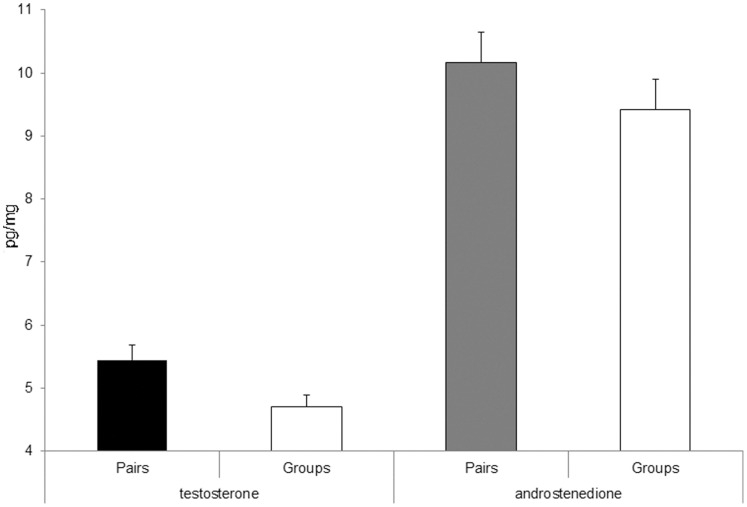
First eggs yolk androgen levels were lower for clutches raised with helpers than for clutches raised in pairs (means and SE are shown). This was significant for testosterone but only marginally for androstenedione (A4).

Yolk testosterone concentration varied between 3.40 pg.mg^−1^ and 7.37 pg.mg^−1^. It did not vary linearly with the number of helpers (F_1,22_ = 2.03, P = 0.17) but was affected by breeding group type (Table 4 ) with a 13.58% reduction in testosterone for eggs laid by females in groups with helpers when compared to those in pairs (Fig. 3). Testosterone concentration tended to decrease with clutch size but this was below the significance threshold (Table 4). There were no effects of egg mass, yolk mass, the number of breeding attempts, colony size and rainfall (Table 4).

**Table 4:** Factors affecting yolk testosterone concentrations.

Explanatory terms	F	d.f.	P	Estimate	SE
**Intercept**				**6,426**	**0,958**
**Group type (H)**	**6,396**	**22**	**0,0382**	−**0,71**	**0,281**
***Clutch size***	***3,337***	***22***	***0,0813***	−***0,562***	***0,308***
Egg mass	0,189	21	0,67		
Yolk mass	0,491	21	0,49		
Clutches attempted before	0,187	21	0,67		
Colony size	0,874	8	0,38		
Rainfall	0,316	21	0,58		

Estimates and SE are given for significant (bold characters) and nearly significant (italic characters) explanatory terms included in the minimal model. ‘Group type’ referred to the presence (H)/absence of helpers feeding a given clutch.

**Figure 4:**
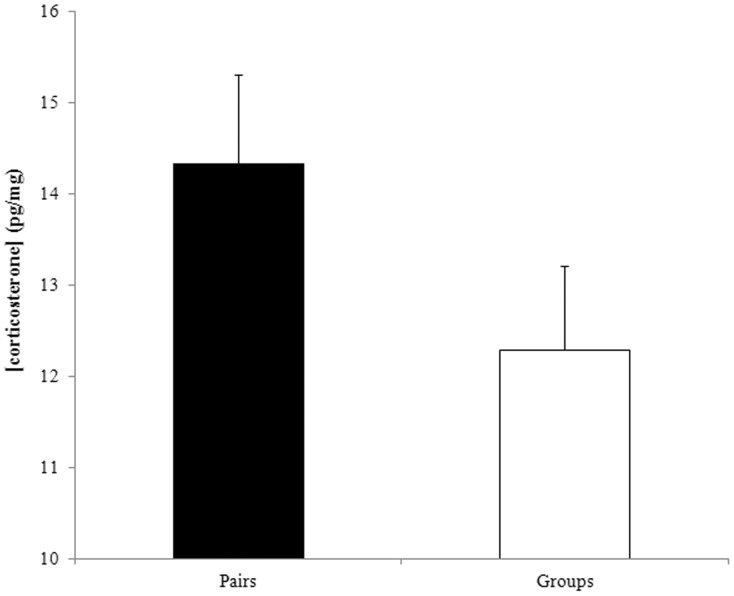
There was a significant decrease in first eggs yolk corticosterone levels (means and SE are shown) between clutches raised in pairs and clutches raised with helpers.

Yolk A4 concentration varied between 5.89 pg.mg^−1^ and 13.32 pg.mg^−1^. There was a non-significant tendency for A4 to decrease with the number of helpers (F_1,22_ = −1.79, P = 0.087, estimate = −0.38±0.21) and to be lower for pairs with helpers than for pairs alone (with a 7.32% reduction in testosterone for eggs laid by females in groups with helpers when compared to those in pairs (table 5, Fig. 4). Yolk A4 concentration also decreased with yolk mass (Table 5); A4 concentrations were higher for second clutches attempted than for the first ones (Table 5). No significant effects were found for the other variables tested (i.e. egg mass, clutch size, colony size and rainfall, all P = 0.38). Yolk A4 concentrations also tended to be lower for pairs with helpers than for pairs without controlling for yolk mass but this tendency was not significant (F_1,22_ = 2.990, P = 0.098, estimate = −1.17±0.68).

**Table 5:** Factors affecting yolk androstenedione concentrations.

Explanatory terms	F	d.f.	P	Estimate	SE
**Intercept**				22,21	2,691
***Group type (H)***	***4,985***	***21***	***0,0732***	−***1,17***	***0,524***
**Clutches attempted before**	**5,520**	**21**	**0,0287**	**1,285**	**0,547**
**Yolk mass**	**21,519**	**21**	**0,0001**	−**18,983**	**4,092**
Egg mass	1,082	20	0,31		
Clutch size	1,075	20	0,31		
Colony size	0,007	8	0,94		
Rainfall	0,604	20	0,45		

Estimates and SE are given for significant (bold characters) and nearly significant (italic characters) explanatory terms included in the minimal model.

### Yolk Corticosterone

Yolk corticosterone concentration varied between 7.31 pg.mg^−1^ and 21.54 pg.mg^−1^. It did not vary with the number of helpers (F_1,22_ = 2.64, P = 0.12) but tended to be lower for the first eggs of females with helpers than for females in pairs (Table 6) with an average reduction of 17.26% (Fig. 4). Corticosterone concentration also decreased with egg mass but no significant effects were found for yolk mass, clutch size, the number of breeding attempts, colony size and rainfall (Table 6). Yolk corticosterone concentration did not vary significantly between group type when egg mass was not in the statistical model (F_1,23_ = 2.30, P = 0.14).

**Table 6:** Factors affecting yolk corticosterone concentrations.

Explanatory terms	F	d.f.	P	Estimate	SE
**Intercept**			**0,0019**	**37,461**	**10,656**
***Group type (H)***	***5,621***	***22***	***0,0538***	−***3,330***	***1,405***
**Egg mass**	**4,749**	**22**	**0,0403**	−**8,987**	**4,124**
Yolk mass	0,067	21	0,80		
Clutches attemptedbefore	0,055	21	0,82		
Clutch size	1,056	21	0,32		
Colony size	1,178	8	0,31		
Rainfall	0,018	21	0,90		

Estimates and SE are given for significant (bold characters) and nearly significant (italic characters) explanatory terms included in the minimal model.

### Effects on Fledging Mass

**Figure 5:**
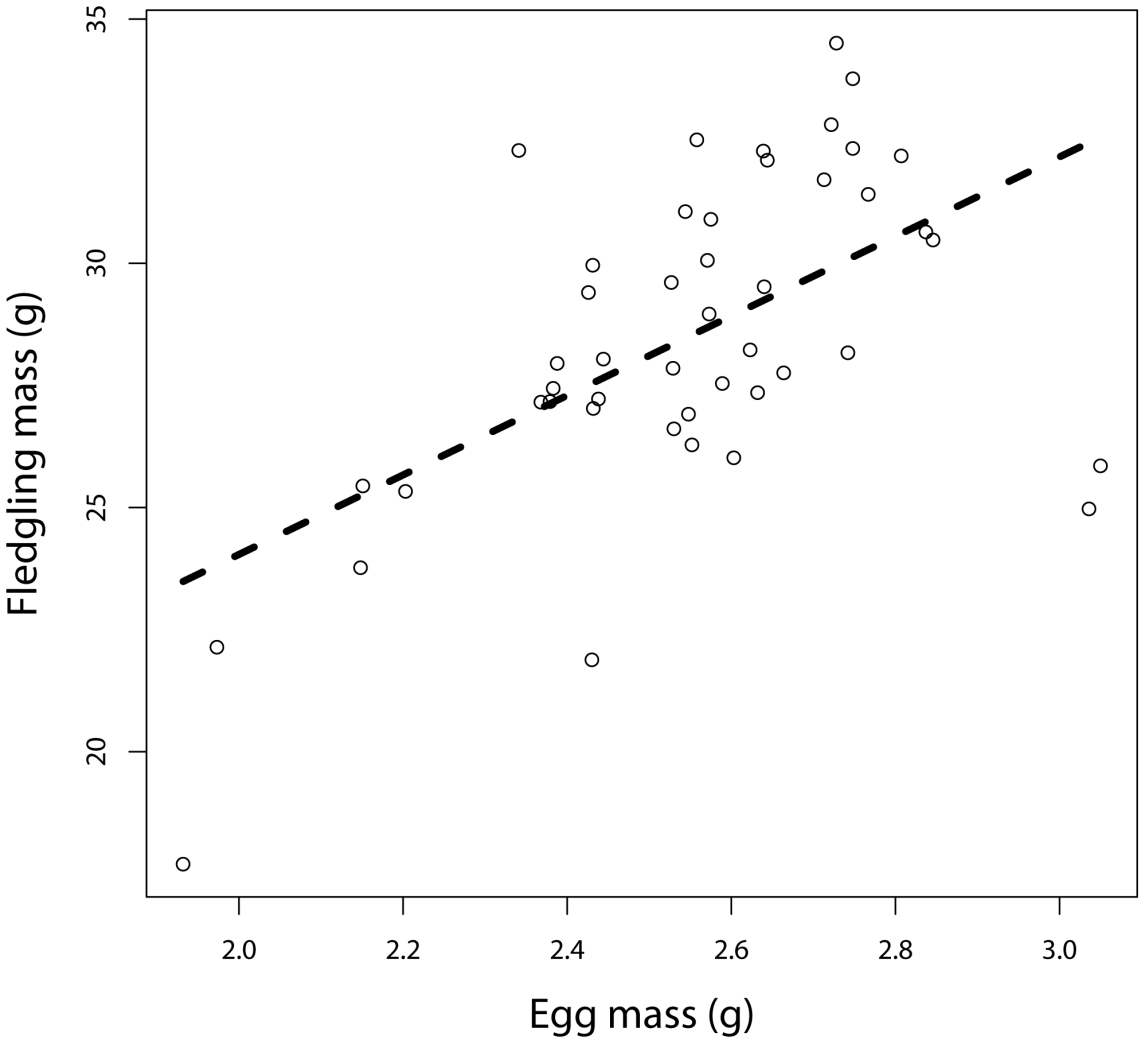
Fledging mass significantly increased in relation to egg mass.

There was no effect of group type (F_1,18_ = 0.11, P = 0.75) or group size on fledging mass (Table 7), regardless of whether egg size was controlled for or not (see Figure S1). The mass of 17 days old chicks decreased with clutch size and hatching order (Table 7). Fledglings were also heavier when coming from heavier eggs (Table 7; Fig. 5). The fledging mass was not affected by colony size, the number of breeding attempts or rainfall (Table 7).

**Table 7:** Factors affecting fledgling mass.

Explanatory terms	F	d.f.	P	Estimate	SE
**Intercept**				**21,673**	**4,690**
**Egg mass**	**12,515**	**16**	**0,0027**	**5,659**	**1,600**
**Hatchling size**	**13,241**	**19**	**0,0017**	−**2,868**	**0,788**
**Hatchling order**	**10,678**	**16**	**0,0048**	−**1,500**	**0,459**
Group size	0,283	18	0,60		
Group size2	0,298	18	0,59		
Clutches attempted before	0,009	18	0,93		
Colony size	0,087	6	0,78		
Rainfall	0,066	18	0,80		

Estimates and SE are given for significant explanatory terms included in the minimal model (bold characters). ‘Group size’ referred to the number of individuals that were feeding a given clutch.

## Discussion

Concurring with four previous studies on other cooperatively breeding species ([47], [48], [50], [51] but see [52]), we found a decrease in sociable weaver egg mass as the breeding group size increased. In addition, we found a negative effect of helper presence on hormonal contents, with lower androgens and corticosterone concentrations in the presence of helpers. To our knowledge, this is the first study to indicate differential maternal allocation of egg hormones in relation to helpers’ presence in an egg-laying species. Although this is a correlative study and experimentation is needed to fully test causality, these results suggest that the environment created by the presence of helpers can influence maternal allocation and offspring phenotypes.

### Maternal Investment in Egg Size and Helper Effects

Maternal load lightening at the egg stage has been found in a broad range of species when a good offspring environment could be anticipated [12], [13], [39], [40]. In cooperatively breeding species, when the breeding groups are already formed before egg laying or live birth, the additional care by the helpers represents a good environment for offspring and should allow females to invest less in their eggs or embryos. This is expected particularly in long-lived cooperatively breeding species where females are likely to keep their dominance status over several reproductive events [41]. In agreement with the previous studies on carrion crows *Corvus corone* [50] and superb fairy-wrens *Malurus cyaneus* [48], sociable weaver females were found to lay lighter eggs as the size of their breeding group increased. In this species egg mass decreased by, on average, 1.67% per additional helper. Egg production is known to be costly in birds ([76], [77] see also [78], [79]). Sociable weavers have protracted breeding seasons, which may last up to nine months under conditions of good rainfall, and have very high nest predation rates (ca. 70% on our study site). As a result, females usually lay several clutches a year (up to 9 clutches have been recorded in a single season; [54]). Under these circumstances, females assisted by helpers are likely to save a considerable amount of energy by producing lighter eggs. Interestingly, this reduction in female investment does not come with a cost for nestlings since we did not find any helper effect on fledging mass, despite a positive relationship between egg and fledging mass. As helpers provide additional food to the brood [54], this suggests that the helpers may compensate for the lower female investment in eggs. However, here we did not find any effect of helpers on fledgling mass even when correcting for egg mass. This might have been a consequence of removing one egg from the brood for analyses of egg contents, since by doing so we artificially reduced the cost of rearing offspring. In addition, the statistical power to detect this relationship might have been limited due to the reduced sample size at fledging, which was a result of nest predation. Additional work is currently underway to test whether the helpers have a compensatory effect on this species.

The energetic savings suggested here may allow females to survive better and therefore increase the number of potential future breeding attempts. Preliminary results suggest an increase in survival for sociable weaver females that have been helped to raise their offspring ([80]; Paquet, Grégoire, Deville, Doutrelant, Covas, in prep). This would indicate that the benefits of helping in sociable weavers may be greater than estimated by previous analyses on the effect of helpers on reproduction which showed that the effect of helpers is mostly positive under adverse conditions [54].

### Maternal Investment in Egg Content

Despite the negative effect of breeding group size on egg mass we did not find the same pattern for the first eggs’ yolk mass. We only found a tendency for a negative quadratic effect of group size on relative yolk mass and did not found any effect of helpers on yolk mass when egg size is not taken into account. As eggs are only constituted by eggshell, yolk and albumen, this suggests that the reduction in egg mass according to group size is mostly due to a reduction in albumen mass. For altricial species, like sociable weavers, eggs are predominantly constituted by albumen [81] which contains on average 71% of the eggs’ proteins [82]. Accordingly, in our study, yolk mass represented on average only 26% of the weight of an egg, the rest being albumen (and eggshell). An energetic model based on Audouin’s Gull’s *Ichthyaetus audouinii* three-egg clutches showed that, for egg formation, the energy-demand peak takes place during the formation of the first egg’s albumen when yolk formation is already completed but females still have to complete forming the yolk of the two following eggs [83]. Therefore, the best way for females to save energy during egg formation is to reduce investment during this peak which can occur by reducing the amount of albumen for the first egg and yolk deposition for second and third eggs [83]. This model explains why first eggs’ yolk mass is much less variable than yolk mass of second or third eggs which is also the case in sociable weavers [74]. Hence, while the reduction of first egg mass with group size seems to result in a reduction of the albumen mass, this may differ for the following eggs in the laying sequence.

This strategy of saving energy by reducing investment in albumen (or, to a lesser extent, eggshell) of the first egg might explain why we found no effect of group size on the amount of yolk carotenoids, which are costly nutrients. Moreover, the effect of helpers may be more complex than a simple expected reduction on carotenoid concentrations. We found that eggs were lighter as the breeding group size increased but on the other hand small eggs may experience a greater oxidative stress and then need more antioxidants like carotenoids to counter it [84].

### Hormones in Presence/absence of Helpers

We found a clear indication of different levels of hormones in relation to helper presence. Females laid first eggs with lower yolk androgen concentrations (significantly lower testosterone and a tendency for lower A4, which was significant before correction for false recovery rates, for both relative and absolute quantities). Corticosterone concentrations are also lower (marginally significant after correction for false recovery rates), but only when we correct for egg mass. Corticosterone is a stress hormone that may be transferred passively [23] and has been found to correlate positively with energy expenditure associated with social status [61]. Hence, females experiencing more stressful or dangerous environments may deposit more corticosterone in eggs [26], [31]. In the cooperatively breeding red-cockaded woodpecker breeding males, but not females, exhibit lower baseline corticosterone levels when assisted by two or more helpers [85]. This was suggested to arise from a reduced workload in the presence of helpers in males at a higher level than females [86]. In sociable weavers both parents reduce their feeding rate in the presence of helpers [54] and in addition helpers are involved in nest chamber defence, nest building and usually roost in the family chamber (Paquet, Covas, Doutrelant, per obs), which may have thermoregulatory benefits [87]. Hence, dominant females may be less stressed when breeding in groups with helpers, which in turn may result in lower circulating corticosterone levels and hence less corticosterone transferred into the eggs. This hypothesis, however, remains to be tested. The higher corticosterone levels transferred in eggs laid by females breeding in pairs may have detrimental consequences for offspring, as too high corticosterone levels were found to reduce nestling growth and begging ability ([25], [27], [30] but see [32]). However, as we did not find a significant lower corticosterone concentration without controlling for egg mass, chicks raised with and without helpers may not experience different circulating corticosterone levels.

The higher level of androgens in eggs produced by females breeding without helpers can be explained by at least three non-exclusive explanations. First, egg androgen levels may be influenced by female breeding condition and social environment. For example in house sparrows *Passer domesticus* the social environment experienced by the breeding female was affected by breeding density and female response to an intruder and this lead to increased yolk testosterone concentration [37]. In sociable weavers social interactions might also play a role if females in pairs are more often involved directly in aggressive interactions. Second, higher allocation of androgens and specifically, testosterone to eggs when breeding without helpers may be a female strategy to manipulate offspring metabolism and begging behaviour. Increasing testosterone levels in eggs has been shown to increase begging behavior and nestling growth [23], [28], [88]. Male sociable weavers bring more food to the nestlings than females [89], and thus, in the absence of helpers, this may contribute to enhance growth as chicks may beg more actively and have higher metabolic activity. In addition, higher testosterone levels may be important to produce more competitive nestlings, since they receive less food when raised by pairs alone [89], yet brood sizes are similar for pairs and groups. Conversely, in the presence of helpers, lower androgens levels should avoid the costs of rearing offspring that beg very actively and/or have enhanced metabolic activity, thus representing an additional way of saving energy [90]. As parents and helpers are likely to respond individually to the begging rate of the chicks [91], the lower testosterone levels in eggs with helpers may be responsible for the load lightening of the feeders. Finally, higher androgen levels could be a strategy to produce more competitive fledglings. For instance, higher concentrations of yolk A4 have been related to the production of more competitive phenotypes in communally breeding and colonial systems [71], [92]. In the present study, A4 tended to be higher in nests without helpers, even if this was not significant. This could increase competitive ability of offspring, increasing their chances to stay in the natal colony and therefore act as helpers in subsequent years. In groups with helpers, competition for staying in the natal groups might be higher when the older, and presumably dominant, helpers are around [93], [94]. Under these circumstances, lower yolk androgens could be beneficial by avoiding conflicts in the group. Further study is needed to test these different possibilities and to relate variation in hormone levels reported here to hormonal, behavioural and fitness variations in nestlings and fledglings.

Here for ethical and practical reasons (i.e. in order to determine group size and composition), we only collected the first egg laid. In order to know if the allocation patterns found here for the first eggs are representative of the female allocation for the whole clutch, a next step would be to collect complete clutches and investigate both yolk and albumen mass and contents in relation to the laying order and helper numbers. A previous study has found variation in yolk mass and carotenoids but not in yolk androgens contents in relation to laying order [95]. Sociable weaver nestlings hatch asynchronously and hence the first-hatched nestling has a higher chance of surviving, which could lead to a compensatory strategy by females. However, it is currently unknown whether this interacts with the presence of helpers. Moreover, the experimental manipulation of the number of helpers before laying remains the only way to fully test the causal effect of helpers on maternal allocation in eggs. As we did not manipulate the number of helpers before laying we cannot completely exclude the confounding factors that may explain the lower investment in eggs in presence of helpers found here. First, ‘good quality’ females could produce more offspring that may become helpers in future broods. However, this confounding effect is unlikely to explain our results since, unlike what was found here, egg mass is expected to be positively correlated with female condition [8]. A second alternative explanation could be that females in groups experience more competition for resources in presence of helpers. This possibility is also unlikely as sociable weavers are non-territorial and the whole colony usually forages communally (authors personal observations; [66]). A competition effect is more likely to occur at the colony level but we controlled for colony identity as a random factor and also included the size of the colony as a covariate in our models and did not find any effects of these variables on egg mass.

In conclusion, our results suggest the existence of differential maternal investment in egg mass and show for the first time that hormonal contents of eggs vary in relation to helper presence in a cooperatively breeding species. These results have two important implications. First, they confirm that modulation of egg mass might be an additional mechanism to consider under the load lighting hypothesis [46] which suggests that helpers are beneficial because they allow parents to save energy for further reproductions. Second, given that the conditions experienced during the developmental stages may exert lifelong influences on adult phenotypes and health [96] the influence of helpers-at-the-nest in cooperative breeders is likely to go beyond the fledgling or independence stages and the effect commonly found on fledging condition or survival. This long-term influence has important implications for understanding the fitness gains of helping. However, the fields of maternal effects and cooperative breeding have so far remained largely apart, and these consequences have not been studied yet. The study of maternal allocation in cooperatively breeding species is therefore a promising research avenue that has the potential to help understanding the high inter- and intra-specific variability on the effects of helpers on key parameters such as reproductive output, survival, dispersal strategies and lifetime reproductive success in cooperative breeders.

## Supporting Information

Figure S1 Non-significant relationships between group size and fledgling mass without and after controlling for egg mass.
(PDF)

Protocol S1 Protocols for egg content analyses.
(PDF)
