# Supplementary material for: Maternal Effects in Relation to Helper Presence in the Cooperatively Breeding Sociable Weaver
Source: PLoS One. 2013 Mar 25;8(3):e59336. doi: 10.1371/journal.pone.0059336 (PMC3607610; doi:10.1371/journal.pone.0059336)
Supplement: Figure S1 — Non-significant relationships between group size and fledgling mass without and after controlling for egg mass. (PDF) [file pone.0059336.s001.pdf]

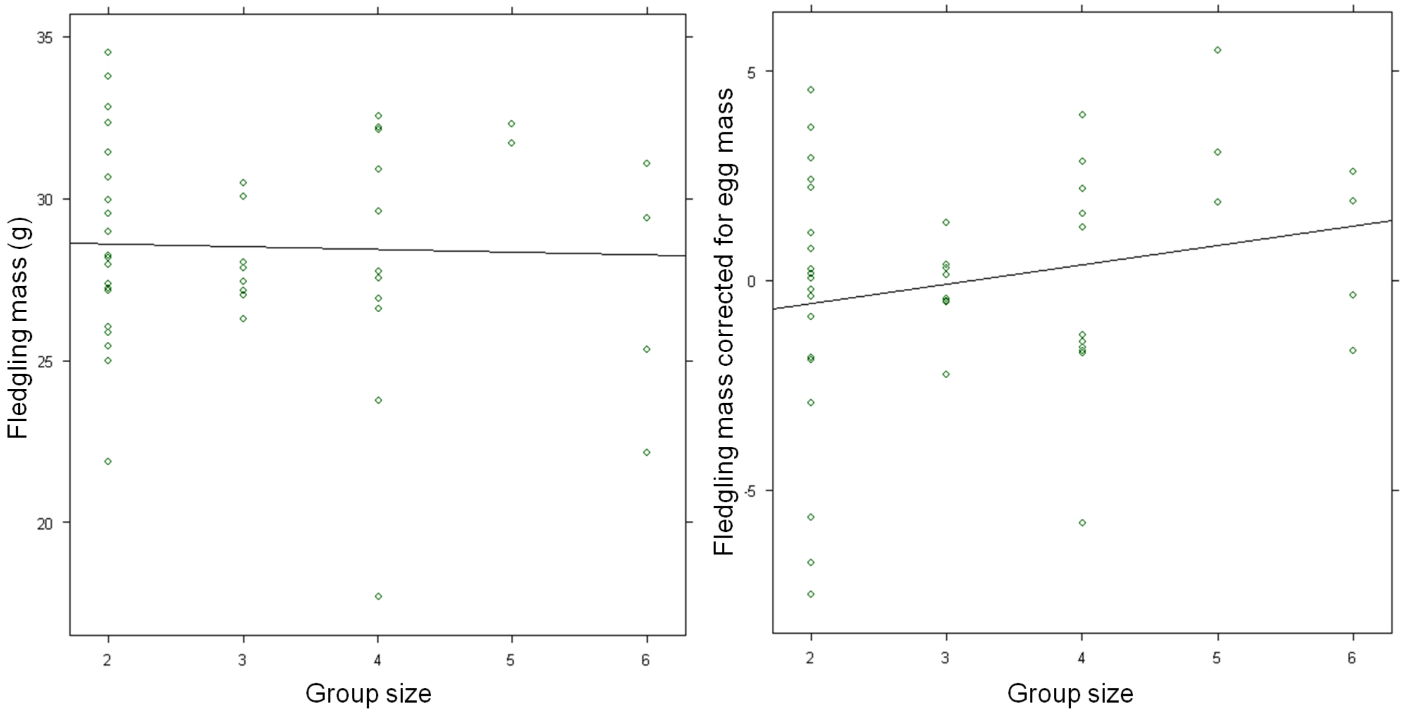

a. **b.**

Non-significant relationships between group size and fledgling mass without (**a.**) and after (**b.**) controlling for egg mass.
