# Supplementary material for: Maternal Effects in Relation to Helper Presence in the Cooperatively Breeding Sociable Weaver
Source: PLoS One. 2013 Mar 25;8(3):e59336. doi: 10.1371/journal.pone.0059336 (PMC3607610; doi:10.1371/journal.pone.0059336)
Supplement: Protocol S1 — Protocols for egg content analyses. (PDF) [file pone.0059336.s002.pdf]

Egg content analyses

The yolks were separated from the albumen while defrosting, weighed at the nearest 0.001g, homogenized and kept at -20°C until analyses.

Carotenoids

For carotenoid extraction 65-75mg of egg yolk was mixed with acetone (1µg of acetone for 0.1mg of yolk). Sample were vortexed, kept one hour at 20°C and then centrifugated at 13000g for 10 minutes. We extracted 3x125µl of supernatant for each sample and determined the optic density (OD) at 450nm in a microplate photometer (Victor[3], Perkin Elmer, France). Commercial solution of lutein (xanthophylls Sigma X-6250) was use to realize a serial dilution and obtain a standard curve to determine the relationship between the OD value and carotenoid concentration in yolk eggs. Inter- and intra-samples variations were respectively 17.55% and 2.61%. We used the mean of the 2 closest values obtained for the 3 replicates as the carotenoid concentration in fresh yolk eggs.

Testosterone, androstenendione and Corticosterone radio-immunoassays

Testosterone , androstenedione and corticosterone were assayed at the Centre d'Etudes Biologiques de Chize with the same procedure. Briefly, 100 mg of each sample were homogenised in 1 mL of distilled water. Steroids were extracted by adding 3 mL of diethyl-ether to 300 µL of the mixture, vortexing and centrifuging (5 minutes at 2000 rpm, at 4°C). The diethyl-ether phase containing steroids was decanted and poured off after snap freezing the tube in an alcohol bath at 38°C. This was done twice for each yolk and the resultant was then evaporated. The dried extracts were redissolved in 900 µL of phosphate buffer and each hormone was assayed in duplicate. 100 µL of extract were incubated overnight with 4000 cpm of the appropriate $_3$H-steroid (Perkin Elmer, US) and polyclonal rabbit antiserum. Anti-testosterone was provided by Dr. Picaper (médecin nucléaire, CHU La Source, Orléans, France), anti-androstenedione by Sigma (US) and anti-11-HS-corticosterone antiserum was

supplied by P.A.R.IS. (France). The bound fraction was then separated from free fraction by addition of dextran-coated charcoal and activity was counted on a tri-carb 2810 TR scintillation counter (Perkin Elmer, US). Cross-reactions of androstenedione antiserum were as follows : 5α androstane-3, 17-dione (67 %), dihydroepiandrostenedione (6 %), testosterone (4.5 %), 11 deoxycorticosterone (<0.001 %), 17β estradiol (<0.001 %), estrone (<0.001 %), progesterone (<0.001%). Cross-reactions of corticosterone antiserum were as follows : cortisone (53 %), 20α hydroxyprogesterone (2.5 %), cortisol (2 %), progesterone (1.3 %), Δ4 pregnen21-ol, 3, 20-dione (0.5 %), aldosterone (0.2 %), 17α hydroprogesterone (0.1%), 1, 3, 5 (10) estratrien 3, 17β-diol (0.1 %), 20β hydroxyprogesterone (<0.03 %). Tests were performed to validate the three hormones assays on egg yolk samples. Inter- and intra-assay variations were respectively 18.01% and 7.27% for testosterone, 20.38% and 4.76% for androstenedione, 30.14% and 7.07% for corticosterone. Testosterone, androstenedione and corticosterone lowest detectable concentrations were respectively 1.59 pg/mg, 2.07 pg/mg and 1.55 pg/mg. Two yolk samples were serially diluted in the assay buffer and their displacement curves were parallel to the standard curve.
